# Visual Detection of Canine Monocytic Ehrlichiosis Using Polymerase Chain Reaction-Based Lateral Flow Biosensors

**DOI:** 10.3390/ani15050740

**Published:** 2025-03-05

**Authors:** Peeravit Sumpavong, Sarawan Kaewmongkol, Gunn Kaewmongkol

**Affiliations:** 1 Department of Veterinary Technology, Faculty of Veterinary Technology, Kasetsart University, Bangkok 10230, Thailand; peeravit.sum@ku.th (P.S.); cvtswt@ku.ac.th (S.K.); 2 Department of Companion Animals Clinical Sciences, Faculty of Veterinary Medicine, Kasetsart University, Bangkok 10230, Thailand

**Keywords:** *Ehrlichia canis*, PCR-based lateral flow biosensor assay (PCR-LFB assay), TaqMan probe-based real-time PCR

## Abstract

A conventional PCR (cPCR) remains an effective molecular technique for the diagnosis of canine monocytic ehrlichiosis. However, agarose gel electrophoresis requires additional time after thermal cycling. In the present study, we developed a PCR-based lateral flow biosensor (PCR-LFB) to detect *Ehrlichia canis* (*E. canis*). Lateral flow strips allow for the simple and rapid detection of PCR products and provide an alternative to gel electrophoresis. The sensitivity, specificity, and detection limit of PCR-LFB were compared to those of TaqMan probe-based real-time PCRs (qPCRs). The PCR-LFB was performed with 5′ 6-FITC and biotin-labeled primers specific to *E. canis*, targeting the *dsb* gene. The detection limit of the PCR-LFB assay was 10^−6^ for the target DNA sequence in a 10-fold dilution of the recombinant plasmid, which is 10 times lower than that of qPCR. Among the confirmed qPCR results in the 30 dog samples, false-positive results were not detected by the PCR-LFB. Compared to qPCR, the sensitivity and specificity of PCR-LFB were 63.6% (95% CI; 42.9–80.2%) and 100% (95% CI; 67.5–100%), respectively. The Kappa value of the PCR-LFB is in moderate agreement with the qPCR (κ = 0.483). Perfect agreement (κ = 1) was observed between cPCR and PCR-LFB. Lower cost and shorter time consumption were demonstrated using PCR-LFB.

## Simple Summary

Agarose gel electrophoresis (AGE) for the detection of PCR products faces several limitations, including low sensitivity for faint bands, time-intensive steps, the risk of contamination, resolution challenges for similar-sized DNA fragments, and health hazards from UV and carcinogenic dyes. The interpretation of the results requires highly experienced technicians. In addition, incompatibility with real-time analysis could reduce convenience utility in clinical settings. These drawbacks highlight the need for faster, more sensitive, and safer alternatives. This study developed the PCR lateral flow biosensor method for the detection of the *Ehrlichia canis dsb* gene, which allows for quicker detection by visually displaying PCR results on a simple test strip.

## 1. Introduction

Canine vector-borne diseases are endemic to tropical and subtropical regions, and evidence of their presence has also been found among canines in temperate areas [1]. Canine monocytic ehrlichiosis (CME) is a tick-borne rickettsial disease that affects dogs and is caused by *Ehrlichia canis* that spread around the world via the arthropod vector, *Rhipicephalus sanguineus* sensu lato (brown dog tick) [2]. *Ehrlichia canis* is a major rickettsial infection that causes febrile conditions, lymphadenopathy, bone marrow suppression, and pancytopenia in dogs [3]. The clinical phase can last from one week to several years. At this chronic stage, the dog can continue to be infected with no clinical signs but with mild thrombocytopenia and hyperglobulinemia; however, ulcerative stomatitis, necrotic glossitis, icterus, and central nervous system signs such as seizures, ataxia, and cervical pain have been more frequently reported in the chronic phase [4,5,6]. The clinical diagnosis of CME is challenging in many dogs with nonspecific indications and signs.

The detection of these pathogens involves a broad range of techniques, including microscopic, antibody-based, and molecular methods [7,8,9]. Molecular diagnostics can detect these pathogens in a wide range of samples and have been demonstrated to be more sensitive than microscopic assays [10]. The conventional polymerase chain reaction (cPCR), which targets several DNA regions, including the 16S rRNA, *trp19*, and *trp36,* has been extensively employed for the diagnosis of *E. canis* infection [11,12,13,14,15,16]. Several PCR modalities have been developed to increase the sensitivity of *E. canis* detection in laboratory procedures [17,18]. Therefore, researchers have focused on quantitative PCR. Quantitative real-time PCR (qPCR) is widely used for the diagnosis of CME. Notably, qPCR, particularly the TaqMan probe-based qPCR approach, offers numerous advantages, including rapid, automated, and extremely sensitive detection and quantification of target DNA in real time [17,19]. However, field tests are hindered by expensive equipment and the need for skilled operators. Therefore, cPCR remains a valuable tool for diagnostic laboratories in remote areas and small veterinary settings in developing countries [20,21]. PCR product detection using cPCR is based on gel electrophoresis, followed by ultraviolet transillumination in the presence of the carcinogenic compound ethidium bromide (EtBr) [22]. However, the use of EtBr is gradually becoming limited and is being replaced by other labels, such as SYBR-based dyes. Although SYBR Green products are safe, some findings indicate a higher level of mutagenicity associated with this compound than with EtBr in bacterial cells exposed to UV radiation [23]. These fragments can be visualized upon exposure to ultraviolet light [24]. A rapid improvement in diagnosis can be achieved by finding new PCR techniques that offer improvements over cPCR; however, these new techniques must closely match the sensitivity and quantification capabilities of qPCR. These transitional techniques often aim to enhance visualization, speed, and ease of use without the need for complex equipment. These new PCR developments have increased during the COVID-19 pandemic [25]. Therefore, the development of new methods for CME diagnosis is necessary to effectively prevent and treat this illness. The increasing demand for simpler signal amplification and product detection methods could make these instruments usable in underequipped laboratories, particularly in developing regions.

The World Health Organization has provided guidelines that are affordable, sensitive, specific, user-friendly, rapid, robust, equipment-free, and deliverable to end users (ASSURED) for the development of diagnostic devices. These recommendations aim to support individuals in developing countries and regions where even moderately priced tests are unaffordable [26,27]. The application of microfluidics in biosensors represents a platform for the development of diagnostic products that adhere to the ASSURED criteria. Furthermore, there are additional benefits such as enhanced portability, reduced sample size requirements, deployment capability in remote areas, decreased power consumption, lower chances of human error, etc. [28].

PCR coupled with lateral flow biosensors (LFBs) can be useful for bacterial detection. Lateral flow (LF) assays are frequently used to rapidly identify and diagnose biological threats, toxins, and infectious diseases. LFBs are important for diagnostic purposes because they are affordable, sensitive, specific, user-friendly, fast, robust, and device-free. Primers and oligonucleotides labeled with small molecules such as biotin or FAM/FITC have been used to detect amplified nucleic acids on an LF strip using tag-specific antibodies. The PCR-LFB assay maintains PCR sensitivity, simplifies electrophoresis, and reduces the cost of real-time detection. LF immunoassays have been reported to detect food-borne pathogens, pathogenic viruses, and bacteria [29,30,31,32].

In the present study, we developed and validated a rapid PCR-LFB assay for the detection of *E. canis* by focusing on a target sequence of the *dsb* gene using 5′ 6-FITC- and biotin-labeled primers. The results on the strip were visualized by the naked eye within 5–10 min of an *E. canis*-specific PCR. This technique has the advantages of being simple to operate and accurate. Additionally, there is no necessity for specialized tools to analyze the results.

## 2. Materials and Methods

### 2.1. Ethical Statement

This study utilized leftover samples from a previous project, as used and published by Sumpavong et al. [33]. The procedures of blood collection and research methodology were approved by Kasetsart University Institutional Animal Care (ACKU61-VET-087) and the Animal Ethics Committee of the Faculty of Veterinary Medicine, Kasetsart University, Bangkok, Thailand.

### 2.2. Blood Sample Collection and DNA Extraction

Thirty blood samples were collected from dogs showing potential clinical signs of ehrlichiosis (pale mucous membrane, petechia, ecchymosis, fever, and anorexia) and harboring ticks at a volume of 2 mL per sample. Variations in blood parameters, including anemia, thrombocytopenia, and pancytopenia, further supported the presence of hematological abnormalities. The whole blood samples were kept in EDTA (4.55 mM/L of blood) and stored at 4 °C until genomic DNA was extracted using an E.Z.N.A. The tissue DNA kit (Omega Bio-Tek, Norcross, GA, USA) was used following the manufacturer’s protocol without modification. The DNA extracts were stored at −20 °C for further analysis.

### 2.3. cPCR Procedures

#### 2.3.1. Amplification of dsb Gene by cPCR and DNA Sequencing

PCR amplification was performed in 20 µL of a 10 x Reaction Buffer (with 15 mM Mg^2+^), 0.2 mM dNTPs, 0.2 µM of each primer (listed in Table 1) without biotin, 2.5 U/µL of YEAtaq II DNA polymerase (Yeastern Biotech, Taipei, Taiwan) and nuclease-free water. The thermal cycling protocols are listed in Table 2. PCR products were verified using 1.5% (*w*/*v*) agarose gel electrophoresis. Gel images were captured using an ultra-slim LED illuminator (Maestrogen, Hsinchu, Taiwan). The PCR products of the expected size were purified using an UltraClean^®^ DNA purification kit (MO BIO Laboratories, Inc., Carlsbad, CA, USA). The purified amplicons were sequenced by First BASE Laboratories (Sdn Bhd, Seri Kembangan, Selangor, Malaysia).

#### 2.3.2. Cloning the dsb Gene

Amplicons of the *dsb* gene were cloned into the pGEMT-Easy vector (Promega, Madison, WI, USA) according to the manufacturer’s instructions. The products of ligation were transformed into *Escherichia coli* DH5α competent cells. Transformants were cultivated, and plasmid DNA was extracted using a GeneJET Plasmid Miniprep Kit (Thermo Fisher Science, Waltham, MA, USA). The concentration of DNA in the pGEMT-*dsb* plasmids was quantified using a Nanodrop 2000c spectrophotometer (Thermo Fisher Science, Waltham, MA, USA). Ten-fold serial dilutions of the pGEMT-*dsb* plasmid were performed, and the resulting dilution series was used to generate a standard curve with triplicate serial dilutions.

#### 2.3.3. TaqMan Probe-Based qPCR of the dsb Gene

The TaqMan probe-based qPCR assay was performed using a CFX 96 system (Bio-Rad Laboratories, Hercules, CA, USA). PCR was performed in a total of 20 µL containing HOT FIREPol Mix Plus (Solid BioDyne, Tartu, Estonia), 1 µM of each primer, and the TaqMan probe. The PCR cycling conditions were as follows: 95 °C for 15 min, followed by 45 cycles of 95 °C for 15 s and 60 °C for 1 min [33]. Fluorescence data were collected using the FAM channel.

### 2.4. Detection of PCR Product Using LF Strip

#### 2.4.1. Nucleic Acid LF Immunoassay

**Figure 1:**
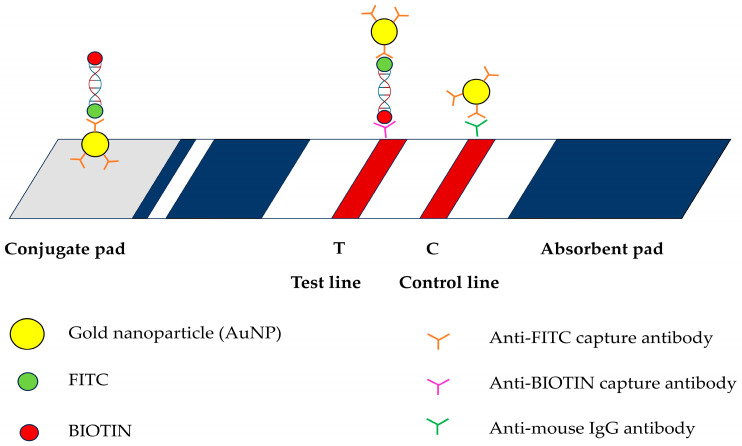
Schematic illustration of lateral flow assay used in this study including gold nanoparticles (AuNP); fluorescein isothiocyanate (FITC); BIOTIN; anti-FITC capture antibody; anti-BIOTIN capture antibody; and anti-mouse IgG antibody.

An LF strip was designed and prepared by Serve Science Co., Bangkok, Thailand. The strip comprised four parts: a sample pad, a conjugate pad, a nitrocellulose membrane, and an absorbent pad. A sample pad was used to load the DNA samples. Gold nanoparticles (AuNPs) labeled with anti-FITC were applied to the conjugate pads. Anti-biotin and anti-mouse secondary antibodies were applied to the T and C lines, respectively, on a nitrocellulose membrane. The absorbent pad facilitated the absorption of the excess solution (Figure 1).

#### 2.4.2. Experimental Procedures of the PCR-LFB

The amplification process for the *dsb* gene followed the same protocol as that for cPCR, with the distinction that the forward primer was modified to include a biotin label. After the PCR process, 10 µL of the biotinylated PCR products was mixed with a FITC-DNA probe (1–2.5 µL) and subsequently heated to establish the appropriate concentration for the analysis of the results. The heating conditions were 95 °C for 5 min to denature double-stranded DNA and 60 °C for 10 min to achieve hybridization, and this was then cooled to 12 °C for 5 min. The hybridized products were loaded onto the sample pad, and four drops (120 µL) of buffer solution were added. The results were observed after 5 min. The T and C lines of the nitrocellulose membrane showing color images were considered positive. A negative result was identified by observing color bands only in the C line.

### 2.5. Determination of Limit of Detection (LoD)

To determine the LoD, the standard plasmid pGEMT-*dsb*, prepared in a previous study, was used [33]. The detection limitations of the PCR-LFB and qPCR assays were compared by 10-fold serial dilutions of the pGEMT-*dsb* plasmid from 10^0^ to 10^−7^ copies/µL.

### 2.6. Testing of PCR-LFB Assay in Naturally Infected Dogs

The PCR-LFB was used to investigate *E. canis* infections in 30 dog samples that were evaluated using both cPCR and qPCR in a previous study [33]. Notably, 2 µL of the extracted DNA was included in the reaction of the PCR-LFB assay to detect *E. canis*. The amplified products were detected using LF strips.

### 2.7. Statistical Analysis

Statistical analyses were conducted using NCSS 2024 (version 24.0.2). The level of agreement between the results obtained using the three methods was assessed using Cohen’s kappa coefficient. The sensitivity, specificity, positive predictive value, negative predictive value, positive likelihood ratio, and negative likelihood ratio were analyzed between a reference standard tool and TaqMan qPCR, cPCR, and PCR-LFB.

## 3. Results

### 3.1. cPCR Amplification of the dsb Gene of E. canis

The cPCR detection of *E. canis* in 30 blood samples, as indicated by the amplification of the *dsb* gene, is presented in Table 3. The DNA sequences of the *dsb* gene amplified from all positive samples were 100% identical to the reference sequence of *E. canis* (GenBank accession number MT659886.1).

### 3.2. cPCR Conditions

#### 3.2.1. DNA Primers and Probes

**Figure 2:**
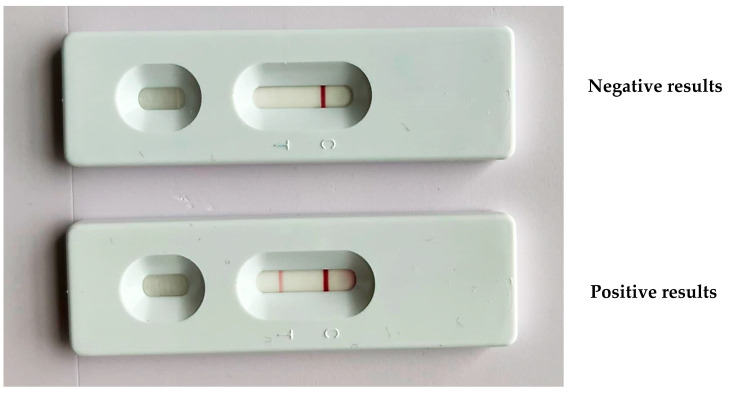
The LF strip device displayed a negative result of PCR products with one band of the control line (C) and a positive result with PCR products that exhibited a band at both the test (T) and control lines.

A *dsb* forward and reverse primer ratio of 1:1 was appropriate to produce a forward biotin PCR-amplified product of 350 bp (Figure S1). A low concentration of the forward primer affected the test results as it yielded negative results for both negative and positive samples. The LF strip produced a clear red band at the test line and accurate results with a 1:1 ratio of forward biotin and reverse FITC *dsb* PCR products (Figure 2).

#### 3.2.2. Effect of the Amount of DNA Probe on LF Strip Testing

**Figure 3:**
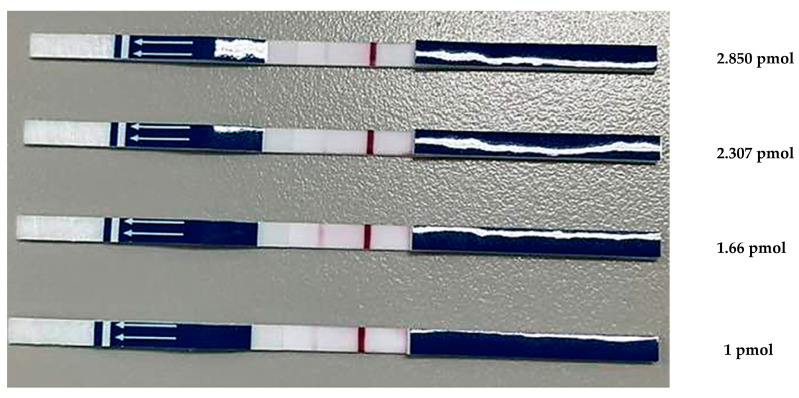
Optimized concentration of DNA probe-FITC. The LF strip device displayed a negative result for PCR products with one band of the control line (C) and a positive result for PCR products that exhibited a band at both the test (T) and control lines.

The DNA probe concentration affected the clarity of the LF strip test lines (Figure 3). An optimal concentration of DNA probe-FITC was required to produce a clear signal. Testing FITC-DNA probe concentrations of 1, 1.66, 2.307, and 2.805 pmol with an LF strip reduced test costs by optimizing the probe concentration. The results obtained at concentrations of 1, 2.307, and 2.805 pmol were characterized by uncertainly, producing inconclusive positive indications. A concentration of 1.66 pmol exhibited a conspicuous and well-defined signal. Therefore, this concentration was used in subsequent experiments.

### 3.3. Detection Limit of PCR-LFB Assay

**Figure 4:**
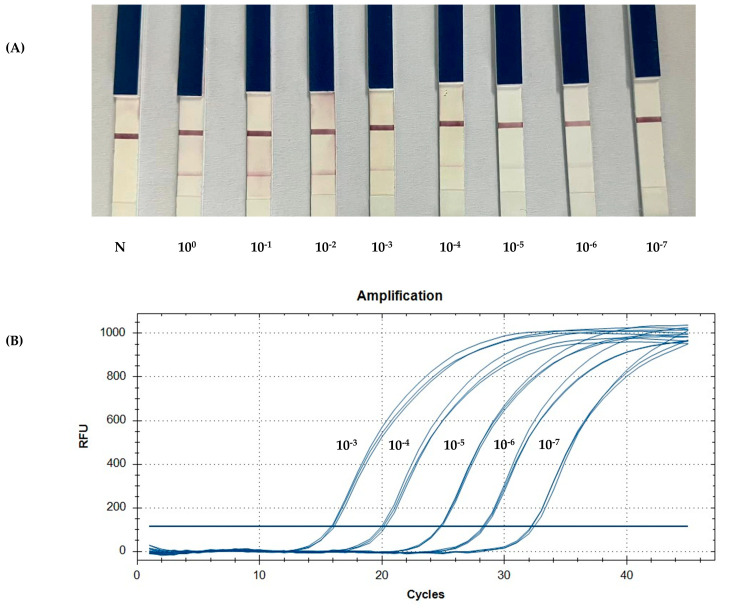
A comparison of the limit of detection between the PCR-LFB and qPCR assays using 10-fold serial dilutions of the known number of copies. (**A**) The detection of the PCR product using an LF strip. (**B**) The standard curve generated by qPCR targeting pGEMT-*dsb*. N: negative control.

To determine the detection limit of the PCR-LFB assay, 10-fold serial dilutions of the known number of copies (10^0^ to 10^−7^ copies/µL) of pGEMT-*dsb* plasmid were used as template DNA. Notably, 2 µL of amplification products was applied on the sample pad of the LF strips to detect the amplification. The qPCR assay demonstrated greater sensitivity than cPCR and PCR-LFB, successfully amplifying target templates diluted to concentrations as low as 10^−7^. In contrast, cPCR and PCR-LFB could only amplify the template diluted to a minimum of 10^−6^ (Figure 4).

### 3.4. Agreement Between PCR-LFB Assay, cPCR, and qPCR

The PCR-LFB assay was performed to detect *E. canis* DNA in blood samples from naturally infected dogs. Positive results were compared among the three tests (Table 3). Of the 30 samples, 22 (73.33%) were detected as positive by qPCR, and 14 (46.67%) were detected as positive by cPCR and PCR-LFB each. Upon comparing the cPCR and PCR-LFB tests with TaqMan qPCR as a reference tool, cPCR and PCR-LFB assays showed a sensitivity of 63.6% (95% CI; 42.9–80.2%) and a specificity of 100% (95% CI; 67.5–100%). The positive and negative predictive values were 100% (95% CI; 78.4–100%) and 50% (95% CI; 28–72%). Based on the qPCR Cq value, the PCR-LFB assay was capable of detecting *E. canis* DNA in naturally infected dogs, with an average Cq value of 30.16. Amplification was visually detected using LF strips within 5–10 min (Figure 4). The results of each molecular test, including cPCR and PCR-LFB, were compared with the qPCR results (Table 4). The PCR-LFB exhibited moderate agreement with qPCR, with κ = 0.483 (95% CI; 0.22–0.74).

## 4. Discussion

The rapid and precise identification of the amplification products of *E. canis* is crucial for the effective diagnosis of CME. This LF strip (Figure 1) is based on antibody interactions. The biotin-labeled PCR-amplified product derived from the target organism was coupled to an FITC-labeled DNA probe during hybridization. The solution was moved across a test strip containing anti-FITC-conjugated AuNPs. Anti-FITC on the surface of AuNPs enables the attachment of AuNPs to DNA, leading to their capture on the nitrocellulose membrane at the anti-biotin test line, resulting in a red band [34]. Additionally, the excess AuNPs interacted with the anti-mouse secondary antibody coating on the control line. Positive results were indicated by the clearly visible red bands on the test and control lines. Negative results were detected when the control line turned red. Rapidity and simplicity are the reasons why the PCR-LFB assay is the preferred choice over traditional diagnostic methods. The cPCR assay requires agarose gel electrophoresis for the endpoint detection of amplicons, limiting its extensive use under field conditions.

In the present study, the ratio of forward to reverse primers was adjusted. Preventing primer self-amplification is necessary. A high level of biotinylated PCR products can produce positive results when no DNA is present. By optimizing the quantity of the forward primer, the probability that the DNA complex from the positive samples binds to the test line and produces a precise result can be determined. Furthermore, the concentration of DNA probes must be optimized. An additional requirement is to adjust the optimal temperature for the hybridization step. Variations in both high and low temperatures may affect the interaction between the probe and the biotin-labeled product.

Using the optimized method, the detection limit of the PCR-LFB assay was 10^−6^ copies (mean Cq value of 32.2) of the target DNA harbored in the recombinant plasmid (Figure 4A). The PCR-LFB assay can identify the presence of *E. canis* in naturally infected dogs with a lower sensitivity than qPCR. The average Cq value of the samples was approximately 30, which was within the detectable range compared to a recombinant plasmid [33]. qPCR effectively evaluated the PCR-LFB assay for the detection of *E. canis*, thereby confirming the efficacy of the device. Although a specificity test was not performed, all the positive samples were identified as *E. canis*. Primer specificity was confirmed by testing against closely related *Ehrlichia* spp. [17].

Based on our results, these three molecular methods could detect *E. canis* in naturally infected dogs with 100% specificity. Compared to qPCR, no false positives were detected by cPCR or PCR-LFB. There was perfect agreement between the PCR-LFB and cPCR assays, as indicated by a kappa value of one. However, agreement with qPCR was only moderate, as evidenced by a kappa value of 0.483 (Table 4). The statistical findings indicate that the PCR-LFB assay exhibits high *E. canis* specificity. However, our technique provided disappointing sensitivity. Therefore, some infected cases may not be detected. Despite this limitation, PCR-LFB remains a promising tool due to its rapid processing time and ease of interpretation of the results, particularly in small veterinary clinics and field settings. Further research on the PCR-LFB should improve sensitivity by finding new target genes or developing a nested PCR that could improve the efficacy of this tool. We evaluated the ability of three PCR methods to detect *E. canis* based on their simplicity and cost-effectiveness. All costs per test and the time consumption for each method were calculated, and the results of each method’s outcome indicators are presented in Table 5. Compared to all three methods, PCR-LFB has the highest cost. Although the cost per test of the PCR-LFB is higher than that of the other tests, costly and specialized equipment or highly skilled personnel are not required. Additionally, the duration required to amplify the nucleic acids and result interpretation is more rapid compared to cPCR.

A typical nucleic acid diagnostic procedure consists of three fundamental steps: the extraction of nucleic acids, the amplification of nucleic acid, and the detection of the amplicons produced. Nucleic acid extraction, the first step in sample preparation, is a critical step that presents considerable challenges for converting laboratory molecular assays into rapid testing. Over the past few years, extensive research has focused on optimizing the procedures for isolating nucleic acids from a range of raw human samples (e.g., blood, saliva, urine, and sputum) and plant tissues [35,36,37,38,39]. In future studies, developing straightforward instrument-free nucleic acid extraction techniques will be crucial for effective and rapid molecular diagnosis in settings with limited resources. This advanced DNA extraction method can accelerate field detection and reduce the need for extensive human intervention.

According to the experiment, the early detection of *E. canis* using molecular assays is crucial to start prompt therapy, which can be lifesaving in various situations. Identifying canine ehrlichiosis involves a combination of clinical signs, hematological analysis, and molecular biology techniques. An alternative PCR-LFB assay can be for *E. canis* detection, which saves money and is easy to use. The effectiveness of PCR-LFB for the detection of *E. canis*, as evidenced by its performance and reliable results, aligns with those of qPCR, supporting its utility in laboratory diagnostics. Compared to routine laboratory diagnostics, the PCR-LFB assay solves the problem of low sensitivity in microscopic observation and eliminates the need for costly electrophoresis equipment. This alternative method is more practical for field detection and small pet clinics. Therefore, the PCR-LFB become a useful tool for technicians and veterinarians in small veterinary settings.

## 5. Conclusions

In this study, PCR-LFB targeting the *dsb* gene successfully diagnosed CME via the visual detection of *E. canis*. The LF strip test maintains PCR sensitivity, simplifies electrophoresis, and reduces the cost of real-time detection. Our approach showed acceptable sensitivity and specificity for detecting dogs that are naturally infected by *E. canis*. The detection process can be performed within 5 min without expensive instrumentation. Utilizing PCR-LFB to detect *E. canis* offers advantages for early detection and further advancements in antigen point-of-care tests for *E. canis* detection. Therefore, this PCR-LFB assay is suitable for field detection in small veterinary clinics.

## Figures and Tables

**Table 1:** The sequence of the primer and probe used for the molecular assay.

Oligo	Sequence 5′–3′	Reference
*dsb*-F	Biotin-TTGCAAAATGATGTCTGAAGATATGAAACA	[17]
*dsb*-R	GCTGCTCCACCAATAAATGTATCYCCTA	
DNA probe	FITC-AGCTAGTGCTGCTTGGGCAACTTTGAGTGAA	

F: forward primer; R: reverse primer; FITC: fluorescein isothiocyanate.

**Table 2:** The thermal cycling protocols for the PCR amplification of the *dsb* gene.

Steps	Temperatures and Time	Number of Cycles
Initial denaturation	94 °C for 1 min	1
Denaturation	94 °C for 30 s	45
Annealing	55 °C for 30 s	
Extension	72 °C for 45 s	
Final extension	72 °C for 10 min	1

**Table 3:** Three PCR assays detected a number of positive dog blood samples.

Number	cPCR	PCR-LFB	qPCR
Positive	14	14	22
Negative	16	16	8

**Table 4:** Comparison of the results of PCR-LFB, qPCR, and cPCR (n = 30).

Test	PCR-LFB		Kappa Value (95%CI)	Degree of Agreement
	Positive	Negative		
qPCR			0.483 (0.22–0.74)	Moderate
Positive	14	8		
Negative	0	8		
cPCR			1	Perfect
Positive	14	0		
Negative	0	16		

CI = confidence interval.

**Table 5:** Cost analysis, the time consumed, and specialized machines for cPCR, PCR-LFB, and qPCR, based on one unknown sample.

Method	Cost per Test	Time-Consuming	Specialized Machine
cPCR	5.31 USD	2.25 h	Thermocycler
PCR-LFB	5.37 USD	2.03 h	Thermocycler
qPCR	2.56 USD	1.40 h	Real-time PCR machine

Cost per test, excluding the thermocycler and real-time PCR machine.

## Data Availability

The original contributions presented in this study are included in the article/Supplementary Materials. Further inquiries can be directed to the corresponding author.

## Supplementary Materials

The following supporting information can be downloaded at https://www.mdpi.com/article/10.3390/ani15050740/s1. Figure S1: Agarose gel electrophoresis (1.2% agarose) of PCR-amplified products using a species-specific primer.
