# Supplementary material for: Visual Detection of Canine Monocytic Ehrlichiosis Using Polymerase Chain Reaction-Based Lateral Flow Biosensors
Source: Animals (Basel). 2025 Mar 5;15(5):740. doi: 10.3390/ani15050740 (PMC11898506; doi:10.3390/ani15050740)
Supplement: Supplementary file 1 [file animals-15-00740-s001.zip › animals-3419800-supplementary.pdf]

Supplement information

Visual Detection of Canine Monocytic Ehrlichiosis Using Polymerases Chain Reaction based Lateral Flow Biosensor

Peeravit Sumpavong [1], Sarawan Kaewmongkol [1] and Gunn Kaewmongkol [2,*]

[1] Department of Veterinary Technology, Faculty of Veterinary Technology, Kasetsart University, Bangkok 10230, Thailand; peeravit.sum@ku.th (P.S.); cvtswt@ku.ac.th (S.K.)

[2] Department of Companion Animals Clinical Sciences, Faculty of Veterinary Medicine, Kasetsart University, Bangkok 10230, Thailand

***** Correspondence: fvetgunn@ku.th (G.K.)

Supplementary Information: Figure of agarose gel electrophoresis

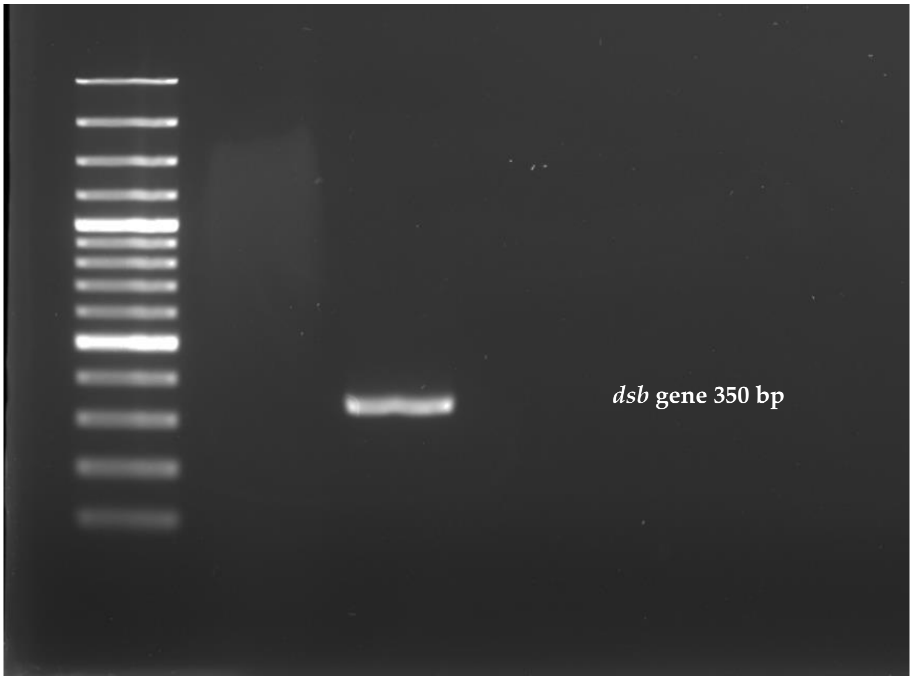

Supplementary Figure S1: Agarose gel electrophoresis (1.2% agarose) of PCR amplified products using species-specific primer.
